# Pathogenic factors associated with development of disseminated intravascular coagulopathy (DIC) in a tertiary academic hospital in South Africa

**DOI:** 10.1371/journal.pone.0195793

**Published:** 2018-04-12

**Authors:** Elizabeth S. Mayne, Anthony L. H. Mayne, Susan J. Louw

**Affiliations:** 1 Department of Molecular Medicine and Haematology, Faculty of Health Sciences, University of the Witwatersrand, Johannesburg, South Africa; 2 National Health Laboratory Service, Johannesburg, South Africa; Maastricht University Medical Center, NETHERLANDS

## Abstract

**Introduction:**

Disseminated intravascular coagulopathy (DIC) is a thrombotic microangiopathy arising from consumption of both coagulation factors and platelets. DIC is triggered by a number of clinical conditions including severe infection, trauma and obstetric complications. Early diagnosis and treatment of the underlying condition is paramount. A high clinical index of suspicion is needed to ensure that patients at risk of developing DIC are appropriately investigated.

**Methods:**

In order to establish the clinical conditions most frequently associated with DIC, we reviewed all DIC screens received at a tertiary hospital in Johannesburg, South Africa over a 1 year period.

**Results:**

The commonest clinical condition associated with DIC in our population was infection with 84% of patients infected with an identified pathogen. The most frequently diagnosed pathogen was HIV followed by *Mycobacterium tuberculosis* and other bacterial infections. In the majority of cases, bacteria were isolated from blood cultures. In 47 patients, HIV was the only pathogen which could be isolated. A relative risk ratio of 2.73 and an odds ratio of 29.97 was attributed to HIV for development of a DIC. A malignancy was present in 51 of the patients of which approximately 60% had co-existing infection. No cause could be attributed in 30 patients.

**Conclusion:**

Infection was identified in the majority of the patients diagnosed with DIC in this study. HIV showed the highest relative risk ratio of all pathogens although previous studies have not suggested that HIV was strongly associated with DIC. In almost half of the HIV infected patients, there was no other pathogen isolated despite extensive investigation. This suggests that HIV has a strong association with the development of DIC, warranting further research into the relationship between HIV and disseminated microvascular thrombosis.

## Introduction

Disseminated intravascular coagulopathy (DIC) is a syndrome of dysregulation of the haemostatic pathways associated with formation of small vessel microthrombi and consumption of platelets and coagulation factors. [1,2] DIC has been associated with a number of clinical conditions including severe infectious disease and sepsis, [3] malignancy,[4] trauma [5] and obstetric complications [6]. The treatment of DIC consists predominantly of managing the underlying disease process and providing supportive treatment for the coagulopathy.[2, 7, 8] Diagnosis of DIC is made using both clinical and laboratory criteria and a number of scoring systems is available. Parameters measured assess the consumption of coagulation factors (prothrombin time, activated partial thromboplastin time, thombin time and fibrinogen levels), anticoagulant activity (antithrombin) and platelet numbers.

Fibrin degradation products (D-dimers) are also included which demonstrate ongoing fibrinolysis (in the context of widespread clot formation and breakdown). [8] Critically, a diagnosis of DIC should be made in the context of an appropriate clinical trigger. [2,8]

The mainstay of treatment of DIC is resolution of the clinical trigger. In patients who are bleeding or who require invasive procedures (like surgery), replacement of depleted coagulation factors and platelets may be indicated. This replacement therapy is monitored using the PTT and aPTT and the platelet count. [2]

The mechanism underlying the development of DIC is not fully understood. Inflammatory stimuli result in endothelial activation with a change from an anticoagulant to a procoagulant surface. [3, 9] This potentiates the formation of small thrombi which eventually consume coagulation factors and platelets resulting in a bleeding diathesis. Early compensatory mechanisms may delay recognition of this process and it is important for clinicians to have a high index of suspicion in the correct clinical context.[4, 9]

Currently, there are little data regarding the aetiology of DIC in South Africa. This is of concern in a setting with a high prevalence of infectious diseases including human immunodeficiency virus (HIV). Early and accurate identification of potential high risk conditions would assist in risk stratification and may assist in creating algorithmic approaches for these patients. [10] In particular, the impact of HIV and *Mycobacterial tuberculosis* infection on the risk of developing DIC is poorly understood.

In order to assess the risk factors for development of these conditions, we conducted a retrospective survey of all DIC screens submitted for analysis to the coagulation laboratory at Charlotte Maxeke Johannesburg Academic Hospital, an academic hospital in Johannesburg South Africa over a 1 year period.

## Methodology

This study received ethics clearance from the Human Resource Ethics Committee of the University of the Witwatersrand (Clearance number: M160839). Participants were assigned a study number and all data were anonymised. All sequential DIC screens received at the laboratory over the period between May 2015 and April 2016 were included in the analysis (n = 401).

All coagulation testing was conducted on a STAGO EVO-R analyser (Cedex, France) on citrate plasma and the platelet count was assessed using a Siemens Advia 120 Haematology analyser (Erlangen, Germany). Where time-to-clot assays were prolonged, a manual correction using normal factor pool was performed. The following data parameters are assessed routinely on DIC panels: Activated Partial Thromboplastic Time (aPTT) in seconds, Prothrombin Time (PT) in seconds, D-dimer levels in g/dl, Antithrombin levels as a percent, Thrombin Time (TT) in seconds and platelet count. Although not allocated points in the ISTH scoring system, antithrombin levels have been utilised by other scoring systems (like the JSTH scoring system) [11] to indicate consumption of anticoagulant factors. Thrombin time has been used historically in our centre to exclude the presence of heparin contamination. D-dimer cut-off levels for moderate and strong increases were set at 0.25-1mg/L and greater than 1mg/L respectively in accordance with previously calculated interquartile ranges. [2]

Information on all screens was accessed from the laboratory information system. All screens were assessed for completeness of data and were assigned a score based on the ISTH DIC scoring system (Table 1). A score of 5 or greater was considered positive for the presence of a DIC. Screens lacking results for D-dimer levels, fibrinogen, PT or platelet count, were excluded from the analysis. Additional clinical data were retrieved from the laboratory information system including the results of bacteriological cultures, histological analysis, viral testing and biochemical testing.

**Table 1:** ISTH DIC scoring system [10].

Laboratory Parameter	Point allocation
**Platelet Count**	
> 100 x10^9^/l	0
50-100x10^9^/l	1
<50x10^9^/l	2
**Increase in Fibrin Markers**	
No change	0
Moderate rise (> 0.25-1mg/L)	2
Strong rise (> 1mg/L)	3
**Prothrombin Time Prolongation**	
3 seconds or less	0
> 3 seconds but < 6 seconds	1
Greater than 6 seconds	2
**Fibrinogen Level**	
Greater than 1 g/l	0
Less than 1 g/l	1

Statistical analysis was conducted using the STATA statistical package (version 14.2 Statacorp, Texas, USA). Relative risk and odds ratios were calculated for all possible DIC triggers utilising the following formulae: Relative risk $\frac{(DIC\;positive\;given\;disease)/(DIC\;positive\;given\;disease\;positive\;and\;negative}{\left(DIC\;negative\;given\;disease)/(DIC\;negative\;given\;disease\;positive\;and\;negative\right)}$ and Odds Ratio: $\frac{\left(DIC\;positive\;given\;disease)/(DIC\;positive\;given\;no\;disease\right)}{\left(DIC\;negative\;given\;disease)/(DIC\;negative\;given\;no\;disease\right)}$.

## Results

Over the study period, 198 of the 401 patients fulfilled the ISTH diagnostic criteria for a diagnosis of DIC. The median age of the patients at the time of presentation with a DIC was 37 years (range 1 month -76 years) with a female to male ratio of 1.2:1. In the majority of patients (84.3%), infection was present at the diagnosis of DIC. The most prevalent pathogen was HIV which was present in 56.6% of these patients. Bacterial infection (excluding mycobacterial infections) was diagnosed in 49 patients and 20 of these had underlying HIV infection. Mycobacterial infection was present in 28 patients, all of whom were HIV co-infected. Malignancy was present in 25.8% of the patients of whom 60% (30) also had co-existing infection.

The most prevalent bacterial infection in the patients was Mycobacterium tuberculosis which was diagnosed in 28 patients followed by *Klebsiella pneumoniae* which was cultured in 12 patients. (Table 2).

**Table 2:** Bacteria and mycobacteria identified at time of DIC diagnosis with site of culture and HIV status.

Organism identified	Number of patients and site of positive test	HIV status of patient at time of culture			Total
		Negative	Positive	Untested	
*Mycobacterium tuberculosis*	Blood (n = 8), Sputum GeneXPert (n = 18) Sputum culture (n = 1), Trephine (n = 1)	0	28	0	28
*Klebsiella pneumoniae*	Blood (n = 7), Urine (n = 5)	6	3	3	12
*Enterococcus faecalis*	Blood (n = 5), Cerebrospinal fluid (n = 1)	3	2	1	6
*Acinetobacter baumanni*	Blood (n = 4), sputum (n = 1)	4	1	0	5
Coagulase negative staphylococcus	Blood (n = 4), sputum (n = 1)	0	4	0	4
*Escherichia coli*	Blood (n = 1), urine (n = 4)	1	0	0	1
*Staphylococcus aureus*	Blood (n = 4)	0	4	1	5
*Pseudomonas aeruginosa*	Blood (n = 1), Cerebrospinal fluid (n = 1) Tracheal aspirate (n = 1), pus (n = 1),	3	1	1	5
*Streptococcus pyogenes*	Blood (n = 3)	0	2	1	3
*Salmonella spp*.	Blood (n = 4)	0	3	1	4
*Streptococcus pneumoniae*	Blood (n = 1), Cerebrospinal fluid (n = 1)	0	2	0	3
*Neisseria meningitis*	Blood (n = 2)	0	2	0	2
*Providentia spp*	Blood (n = 2)	0	0	2	2
*Haemophilus parainfluenza*	Sputum (n = 1)	0	1	0	1
*Streptococcus anginosus*	Blood (n = 1)	0	1	0	1
*Proteus mirabilis*	Urine (n = 1)	0	1	0	1
Mixed Growth	Urine (n = 3), Blood (n = 1)	1	2	1	4

Fungal infections were demonstrated in 12 patients and a further 4 patients had evidence of a suspected fungal infection (an elevated beta-D glucan level) although blood and urine cultures failed to demonstrate a fungal pathogen (Table 3). Only 4 patients had evidence of fungal septicaemia.

**Table 3:** Fungal pathogens identified at the time of DIC diagnosis.

Fungal pathogen	Site of identification and number of patients	HIV status			Total
		Number			
		Negative	Positive	Untested	
*Candida spp*	Urine (3), Sputum (1), Cervical (2) Blood (1)	1	5	1	7
*Candida albicans*	Blood (1), Urine (1)	-	1	1	2
*Candida parapsilosis*	Blood (1)	1	-	-	1
*Cryptococcus neoformans*	Blood (1), Cerebrospinal fluid (1)	-	2	-	2
High B-D glucan		-	4	-	4

Malignancies were present in 51 patients at the time of diagnosis. Haematological malignancies were the most commonly identified (n = 37) with Acute myeloid leukaemia predominating. The commonest solid organ malignancy identified was squamous carcinoma of the cervix which was present in 5 patients at diagnosis (Table 4).

**Table 4:** Underlying malignancies present in patients with diagnostic features of a DIC.

Primary diagnosis	Staging (Solid organ malignancies)	World Health organisation subtyping (Haematological malignancies)	HIV status			Total
			Number of patients			
			Negative	Positive	Unknown	
**Haematological Malignancies**						
Myelodysplastic syndrome		Myelodysplastic syndrome with excess blasts	2			2
Acute myeloid leukaemia (AML)		AML with recurrent translocation t(15;17) n = 5 AML with recurrent translocation t(8;21) n = 3 AML NOS, Acute monoblastic n = 1 AML not otherwise specified n = 6	4	7	4	15
Chronic Myeloid leukaemia		CML with BCR-ABL1	-	-	2	2
B-cell Acute Lymphoblastic leukaemia (ALL)		B-cell ALL, not otherwise specified n = 2	1	-	1	2
Acute leukaemia		Not subtyped	2	-	1	3
Diffuse Large		Gastric (n = 1), Plasmablastic (n = 1), NOS (n = 1)	-	2	1	3
B-cell lymphoma						
B-cell Lymphoproliferative disorder		Not subtyped	-	1	-	1
Hairy cell leukaemia		Hairy cell leukaemia	1	-	1	2
Castleman’s Disease		Castleman’s Disease	1	-	-	1
Monoclonal gammopathy of uncertain significance			1	-	-	1
Hodgkin Lymphoma		Classical (n = 1), Nodular sclerosing HL (n = 1) HL NOS (n = 1)	-	3	-	3
Plasma cell myeloma			1	1	-	2
**Solid Organ Malignancies**						
**Adenocarcinoma**						
Breast Ductal	Stage 4		-	-	1	1
Ovarian	Stage 4		-	1	1	2
Prostatic	Stage 4		1	-	1	2
**Squamous carcinoma**						
Cervical	Stage 1 (1) Stage 2b (1) Stage 4 (3)		1	3	1	5
Oesophagus	Stage 4		-	1	-	1
No identifiable primary	Stage 4		-	-	2	2
**Sarcoma**						
Kaposi Sarcoma	Stage 4		-	1	-	1

HIV infection, itself, had the highest relative risk ratio for all possible triggers of 2.738 (95% confidence limits 2.32–2.32) followed by bacterial infection. The Odds ratio for development of DIC was highest for mycobacterial infection (Odds ratio of 35.005, 95% confidence limits of4.7–259.66) followed by HIV and then non-mycobacterial bacterial infections (Table 5).

**Table 5:** Relative risk ratios for suspected triggers for DIC.

	Bacterial (non-mycobacterial)	*Mycobacterium tuberculosis*	Malignancy	HIV
**Odds ratio**	27.2199	35.0059	22.7478	29.9776
95% lower confidence limit	8.3579	4.7193	6.9607	12.7020
95% upper confidence limit	88.6490	259.6629	74.3400	70.7492

**Relative risk ratio**	2.3110	2.1335	2.2310	2.7387
95% lower confidence limit	2.0104	1.8738	1.9414	2.3257
95% upper confidence limit	2.6565	2.4292	2.5638	3.3257

## Discussion

Over the study period, 198 patients fulfilled the ISTH DIC criteria for diagnosis of a DIC. As anticipated, the commonest potential trigger for the DIC identified in these patients was infection with a positive assay for viral, mycobacterial, fungal or bacterial infection in approximately 84% of the patients.

The commonest pathogen identified was HIV. HIV has been reported as a potential cause of DIC although this has generally been in association with an opportunistic or co-existing infection or malignancy. [12, 13] HIV infection has been reported more commonly as a trigger for other microangiopathic haemopathies specifically thrombotic thrombocytopaenic purpura (TTP). [14, 15] Early studies suggest that DIC is an uncommon complication in HIV. HIV is, however, associated with an increased risk of endothelial dysfunction and activation. [16] The increased risk of dysfunctional clotting in HIV-infected individuals has been attributed to a number of risk factors including low levels of the anticoagulant factor protein S [17], activating antibodies, reduced processing of procoagulant factors like von Willebrand factor and low level chronic inflammation. [18–20] Although a large number of HIV-infected patients (43.8%) in this study were co-infected with an additional pathogen and 16 had an underlying malignancy (14%), in 47 patients (42% of all HIV infected patients), no additional pathology could be found despite extensive investigation and repeated cultures.

The ISTH DIC diagnostic score allocates points for thrombocytopaenia, elevated fibrin degradation products (D-dimers), prolonged PT and fibrinogen. Elevated D-dimer levels have been reported in HIV patients, particularly in the context of uncontrolled viraemia. [21] The platelet count is often decreased in these patients for a number of reasons including autoimmune platelet destruction, marrow infiltration and HIV-associated dyshaemopoiesis.[22] Chronic infection with liver synthetic dysfunction in these patients could also result in perturbation of the coagulation cascade and decreased fibrinogen. [23] These factors may impact the laboratory analyses and the diagnosis of DIC in these patients may not reflect a pure consumptive coagulopathy. The clinical presentation is, however, indistinguishable with high morbidity and mortality and the treatment for these patients includes treatment of the underlying cause. This would include treatment of all infections with initiation of chemotherapy for tuberculosis and antiretroviral therapy where appropriate.

In 30 patients, no clear trigger for the underlying DIC could be ascertained. It is suspected that, in some of these patients, investigation was limited owing to early mortality. HIV testing can only be performed after counselling and consent and it was beyond the scope of the study to test the patients retrospectively. The clinical features were, in some cases, suggestive of underlying immunosuppression.

This study has a number of limitations. Results were assessed retrospectively and no additional testing was performed. Subjects could only be identified if a sample was submitted for DIC screening and it is possible that not all patients in the hospital were appropriately investigated for DIC. This could result in bias as there is an overrepresentation of DIC screens from intensive care units and internal medicine wards where the clinical suspicion for DIC is high. There was an underrepresentation of screens from the surgical, trauma and obstetric departments where DIC may be consequently under-diagnosed. It was not possible to determine whether patients fulfilled the criteria for diagnosis of sepsis as commonly used scoring systems require clinical data. [24] The study contained a small number of participants. Finally, it was not possible to follow patients up and the final clinical outcome of the DIC could not be evaluated. The study does, however, highlight the common triggers for DIC in our setting and may alert clinicians to ensure appropriate investigation and treatment.
